# A bibliometrics visualization analysis of active packaging system for food packaging

**DOI:** 10.1016/j.heliyon.2023.e18457

**Published:** 2023-07-20

**Authors:** Andi Dirpan, Andi Fadiah Ainani, Muspirah Djalal

**Affiliations:** a Department of Agricultural Technology, Hasanuddin University, Makassar 90245, Indonesia; b Center of Excellence in Science and Technology on Food Product Diversification, Makassar, Indonesia; c Research Group for Post-Harvest Technology and Biotechnology, Makassar 90245, Indonesia

**Keywords:** Active packaging, Food packaging, Shelf life, Antimicrobial, Bioplastic

## Abstract

This bibliometric study includes publications on the use of active packaging in food packaging from 2000 to 2021. The number of research related to this study tends to increase annually with an annual growth rate of 23.76%, totaling 857 articles. In this study it was found that the most influential countries in the field of Active Packaging are Spain, China, and Brazil. Moreover, the *International Journal of Biological Macromolecules* and Nerín are the most prolific journal and author in scientific publications, respectively. Active packaging, food packaging, and antimicrobial are often used based on the total link strength out of the 1,775 keywords. The keyword analysis based on time found new terms that are being studied by many researchers, namely, bioplastics as environmentally friendly packaging, based on polysaccharides and nanoparticles, which have the potential to be developed or collaborated for breakthroughs. Therefore, the use of active packaging shows a promising trend for the packaging industry in the future.

## 1 Introduction

Packaging is one of the important components of the food-processing sector because it protects food and affects the consumer's first impression of a food product. It serves three main purposes: protection, conveying information, and transportation [1]. Thus, packaging greatly affects consumers' decisions to buy or not buy a product. Recently, the consumer demand for easy to make food with long shelf life has increased [2,3]. This resulted in several scientific publications and studies to describe new technologies in food packaging, particularly active packaging. Active packaging not only protects but also extends the shelf life of a product by maintaining or improving the sensory, safety, and quality of perishable food products such as meat, bakery, dairy, fruits and vegetables, and others [[4], [5], [6]]. This active packaging has the concept of adding certain components that can release or absorb substances from or into the packaged product.

Generally, active packaging is divided into non-migrating and active-release packaging. Non-migrating packaging is designed to remove unwanted components from the environment without direct contact with the product. Meanwhile, active-release packaging uses emitters that allow controlled migration of the desired substance to directly affect the product [7]. Non-migrating active components tend to function as ethylene absorbers, oxygen generators, and moisture scavengers [[8], [9], [10]], whereas active-release components function as carbon dioxide generators, antimicrobials, and antioxidants [[11], [12], [13], [14]].

A report by Grand View Research Company (San Francisco, USA) [15] estimated that by 2024, the active packaging market will generate $6 billion in the USA. Undeniably, active packaging applications have increased in various countries. Therefore, assessing the study scope that tends to offer data about current projects, trends, scientific collaborations, and their effects is important. This study uses bibliometric techniques to assess developments and trends in the field of active food packaging. This technique can be used to map trends related to authors, institutions, and countries and identify gaps in specific areas, for which bibliometric analysis is highly preferred [16,17].

To the best of our knowledge, many previous bibliometric studies have focused on food packaging, such as the use bionanocomposite materials as packing materials [18], production of cellulose-containing food packaging [19], gelatin packaging/coating to enhance food shelf life [20], intelligent system to monitor and predict packaged food contamination [21], and novel development of materials for food packaging [22]; however, they do not specifically discuss active packaging. Active packaging can be an important part of the food-processing industry to protect and preserve products and meet customer expectations. Thus, knowledge of the developments and trends in the packaging industry, especially in active packaging, given the high consumer demand for safe and quality food, is important.

To better understand the use of active packaging in food packaging, we have collected data from various publications between 2000 and 2021, where in these years various researchers have made massive development in active packaging. This study offers a dense pool of knowledge where researchers can access important data on trends and open questions for further research that can expand the use of active packaging in food packaging to maintain product quality, safety, and shelf life. Based on this, we tried to analyze and explore some sections that we consider interesting for researchers and manufacturers in the field of food packaging: (1) exploring the global trends of active packaging; (2) analyzing the contributions and collaborations of different countries, institutions, and journals to this field, highlighting the most influential papers; (3) analyzing the most productive authors in the field; (4) revealing the research focus through keyword analysis; and (5) identifying the future for active packaging.

## 2 Methodology

### 2.1 Data collection

This study used data extracted from Scopus, a bibliographic resource owned by Elsevier, which provides various functions to support a robust bibliometric analysis [23,24]. Scopus is a popular tool among academics seeking access to high-quality analytical insights [24,25]. This electronic database, which is notably larger than the Web of Science or PubMed and more accurate than Google Scholar, offers extensive coverage of peer-reviewed scientific literature and has an outstanding citation record [26,27]. Scopus was selected as the data source for this review based on its reputation as a comprehensive and reliable repository of scholarly works.

**Fig. 1:**
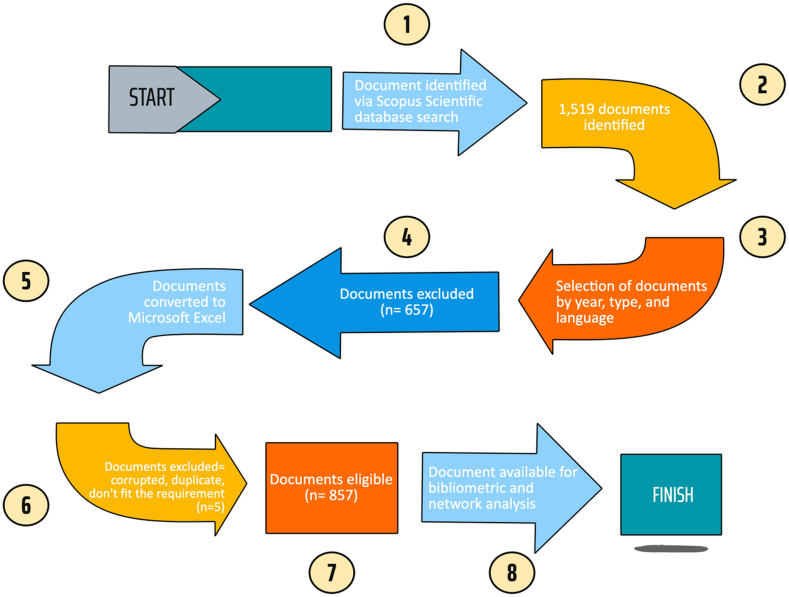
Outline of the search strategy for the bibliometric analysis.

Fig. 1 shows the outline of the search strategy for this bibliometric review. The search method used a topic search including title, abstract, and keywords to seek all related publications. These keywords include TITLE-ABS-KEY (“active packaging”), OR (“active package”), and AND (“food packaging”).

To prevent bias and inconsistencies caused by database changes, data collection was conducted on October 28, 2022, which yielded 1,519 articles. This study focused on publications between 2000 and 2021. The search was limited to English-language publications, articles, publication stage final papers, and source-type journals. Finally, 857 articles were eligible for analysis. Bibliometric data from the selected publications were converted to comma-separated value files. Data were sorted and organized using Microsoft Excel, as follows: (1) authors, (2) title, (3) year of publication, (4) source/journal where the article was published, (5) research institutions, (6) author keywords, and (7) country/region. Before analysis, data were first screened, and corrupted data and duplicates were removed. Some indicators were sorted from the highest rank using the standardized competitive ranking. In particular, for the top 10 journals indicator, the impact factor (IF) was attached. The IF of a journal indicates the average number of annual citations received from recent articles published in that journal [28].

### 2.2 Data Analysis

The OpenRefine application (v. 3.6.1) was employed to manually combine words that have the same meaning. For instance, active package and active packaging were combined. Poly-lactic acid and poly (lactic acid) were also manually combined into PLA. Furthermore, polyvinyl alcohol, poly (vinyl alcohol), and polyvinyl (alcohol) were combined into PVA because the acronym facilitated visualization. Singular and plural forms of the same noun such as packaging materials and packaging material were also combined manually. In addition, VOSviewer (v. 1.6.15.0) was used to create bibliometric graphical mapping and network visualization using the most frequent terms, countries, affiliations, and journals that contribute the most and are interrelated in studies on active packaging, such as those conducted by Refs. [19,29,30]. Meanwhile, RStudio (v. 4.2.1) was used to visualize the analyzed data and plot a global map showing the geographic distribution of articles.

## 3 Results and analysis

### 3.1 Main information about data (2000–2021)

In this study, only those articles published between 2000 and 2021 were included. A total of 857 articles were generated from Scopus, involving 189 journals and 2,941 authors (Table 1). The average number of citations was 45.49, with an annual growth rate of 23.76%, and a total of 39,677 articles were a reference to 857. After 2015, the number of annual publications grew almost exponentially, with the highest recorded in 2021 reaching 176.

**Table 1:** Major information on active packaging for food packaging research from 2000 to 2021.

Description	Results
Main information about the data	
Timespan	2000:2021
Sources (Journals)	189
Documents	857
Annual Growth Rate %	23.76
Document Average Age	5.12
Average citations per doc	45.49
References	39,677
Document contents	
Keywords Plus (ID)	4,556
Author's Keywords (DE)	1,775
AUTHORS	
Authors	2,941
Authors of single-authored docs	11
Authors collaboration	
Single-authored docs	14
Co-Authors per Doc	4.98
International co-authorships %	23.69
Document types	
article	857

### 3.2 Trend in the number of publications

**Fig. 2:**
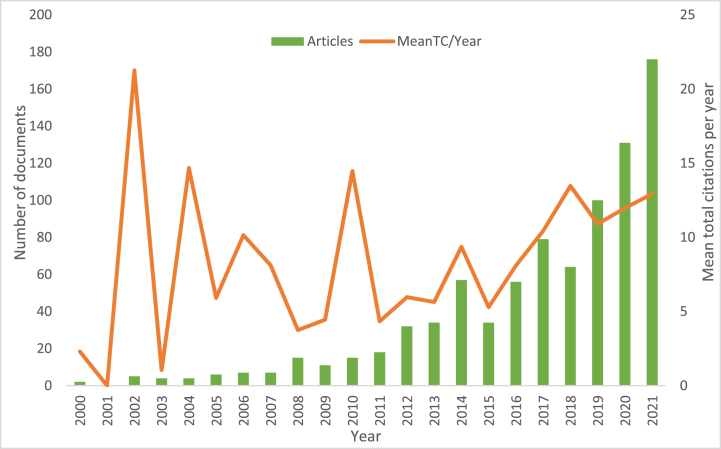
Annual production and average citation per year research on active packaging for food packaging from 2000 to 2021.

Fig. 2 shows the annual development of publications concerning active packaging. Publications between 2018 and 2021 showed a significant increase of approximately 54.9% from 857 total articles. The highest number of publications was observed in 2021 with approximately 176 articles, accounting for 20.5%. An annual growth rate of 23.76% indicated research trends in the use of active food packaging over time. The increase in publications was attributed to the awareness of using a particular packaging that can extend shelf life and maintain product quality. Active packaging provides additional functions to improve food quality and safety [31].

### 3.3 Analysis of the contribution of different countries

**Fig. 3:**
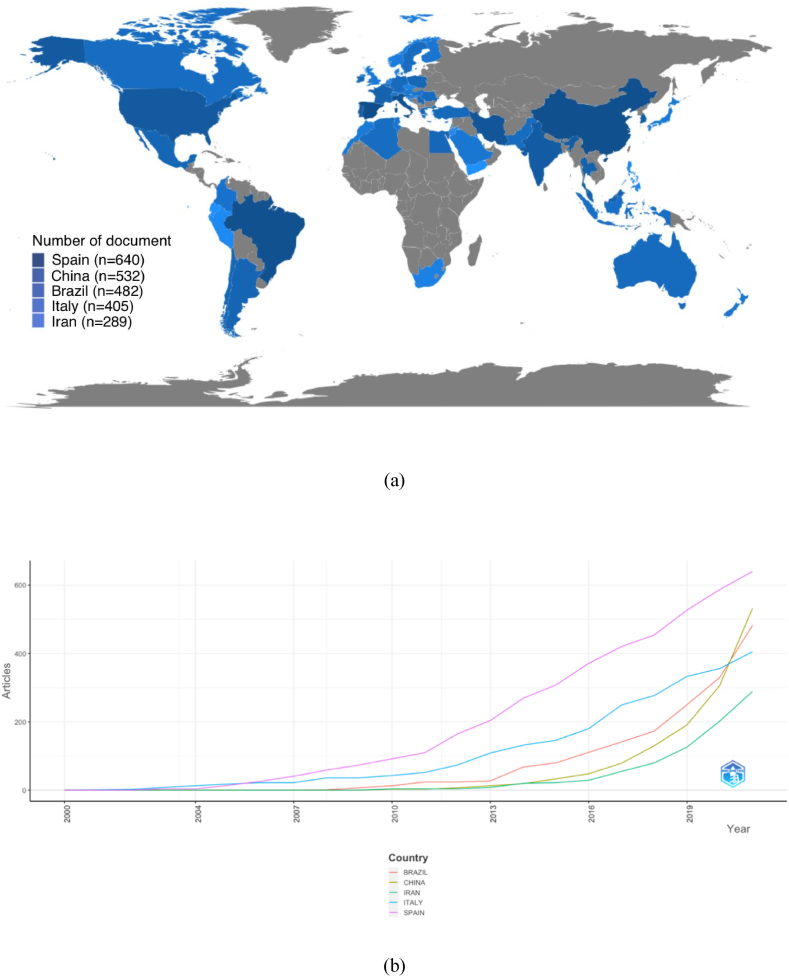
Country scientific production on active food packaging from 2000 to 2021 (a) and the most five country production over time (b).

Countries with the most publications related to active food packaging are shown in Fig. 3a: the darker the blue color, the higher the number of articles published. A total of 58 countries contribute to active packaging studies. Of the top five countries, Italy was the first to publish in 2002 (n = 2), followed by Spain in 2003 (n = 4), Brazil in 2008 (n = 1), China (n = 3), and Iran (n = 4) in 2010. Fig. 3b shows the annual article publication of the top five countries.

In 2021, Spain published approximately 640, which was the highest number of articles related to active packaging, followed by China, Brazil, Italy, and Iran with a total of 532, 482, 405, and 289, respectively. Spain cumulatively contributed to this study out of the top five countries. Spain is a leading country in the study of active food packaging because of its high level of expertise in the food industry and a strong focus on research and development. The food industry in Spain is one of the most important in Europe and is known for its innovation and quality [[32], [33], [34]]. As such, the study of active packaging has been extensively pursued in Spain, given its capacity to enhance food product shelf life and maintain quality, which is of paramount importance for perishable food items, including fresh fruits and vegetables, meat, and dairy products. Furthermore, Spain's rich tradition in food preservation has played a pivotal role in the advancement of active packaging, which is a logical progression of its extensive expertise in this field [35,36].

### 3.4 Analysis of articles in accordance with the journal

Table 2 shows the top publications on “active food packaging.” A total of 189 journals that published 857 articles were analyzed. However, the top 10 publications showed approximately 22% of the total articles produced. The *International Journal of Biological Macromolecules* had the most published articles (n = 84, IF = 8.025), followed by *Food Packaging and Shelf Life* (n = 47, IF = 8.749), and *Journal of Food Science* (n = 44, IF = 3.693). Moreover, the *Journal of Macromolecule Biology* had the highest number of citations with 3,584, followed by *Food Hydrocolloids* and *Food Chemistry* with 3,143 and 2,292, respectively. Of the 10 journals with the most publications, *Packaging Technology and Science* published its first article in 2000, whereas the *International Journal of Biological Macromolecules* and *Food Packaging and Shelf Life* published their first articles in 2014.

**Table 2:** Top-ten journals that published the highest number of articles on the subject.

SCR	Journal	Documents	Total citations	Avg. Cit/doc	Best Rank	IF	PY_start
1st	International journal of biological macromolecules	84	3,584	11.6	Q1	8.025	2014
2nd	Food packaging and shelf life	47	1,673	10.8	Q1	8.749	2014
3rd	Journal of food science	44	1,243	5.1	Q1	3.693	2003
4th	Food chemistry	39	2,292	13.1	Q1	9.231	2007
5th	Journal of agricultural and food chemistry	34	1,923	8.6	Q1	5.895	2005
6th	Food hydrocolloids	32	3,143	15.3	Q1	11.504	2009
7th	Carbohydrate Polymers	29	2,166	16.0	Q1	10.723	2013
8th	International journal of food microbiology	24	1,574	9.4	Q1	5.911	2006
9th	Lwt	21	902	7.3	Q1	6.056	2012
10th	Packaging technology and science	20	631	4.2	Q2	10.723	2000

SCR = rank, IF = impact factor, PY_start = publication year.

### 3.5 Analysis of articles in accordance with the authors

The data analyzed were 857 articles written by 2,941 authors, and the top 10 authors sorted by the most publications related to active food packaging are shown in Table 3. RStudio was used to analyze the productivity and effect of author citations in relation to the h-index. The h-index is an indicator that can measure the contributions and achievements of researchers; however, it cannot be used to evaluate various disciplines [37]. In addition, citations can be used to measure an author's influence on a subject. As presented in Table 3, Nerín had the most publications (n = 26), but only ranked third in the number of citations (1,315 total citations). The first and second places in the number of citations were occupied by Rhim (1,547 total citations) and Gavara (1,358 total citations). The year of publication is one of the criteria that affect how often an article is cited. Rhim and Gavara with the same year of publication (2007) received more citations than Nerín who published in 2005 (2 years earlier).

**Table 3:** Top ten authors who participated the most in studies on “Active Packaging for Food Packaging”.

SCR	Authors	h-index	TC	Documents	Institutions	Country	PY_start
1st	Nerín C	21	1,315	26	Universidad de Zaragoza	Spain	2005
2nd	Gavara R	20	1,358	24	Consejo Superior de Investigaciones Científicas	Spain	2007
3rd	Liu J	18	1,030	19	Yangzhou University	China	2009
4th	Lagaron JM	16	1,087	17	Instituto de Agroquimica y Tecnologia de los Alimentos (IATA)	Spain	2007
5th	Rhim J-W	14	1,547	17	Kyung Hee University	South Korea	2007
6th	Goddard JM	11	293	17	Cornell University	United States	2011
7th	Hernández-Muñoz P	12	845	16	Instituto de Agroquimica y Tecnologia de los Alimentos (IATA)	Spain	2008
8th	Liu, Y	13	704	15	Yangzhou University	China	2017
9th	Decker EA	10	236	11	University of Massachusetts Amherst	United States	2012
10th	Almasi H	9	428	9	Urmia University	Iran	2017

As presented in Table 3, Nerín, who was from Universidad de Zaragoza, Spain, contributed the most to research on active packaging, with 26 articles and 1,315 total citations. In her research, Nerín focused on the use of active ingredients as a source of antioxidants such as cinnamon essential oil, ethyl lauroyl alginate, carob (*Ceratonia siliqua* L.), olive leaf extract, flaxseed oil, ginger essential oil, grape seed essential oil, and rose oil, which can function as antimicrobial substances in active packaging [[38], [39], [40], [41], [42]].

The second-ranked author with an impact on this study was Gavara from Consejo Superior de Investigaciones, Spain, who had 24 articles and 1,358 total citations. In general, Gavara R discussed the effectiveness of antimicrobial activity in the application of essential oils and thymus plants as active packaging. The tested oils include garlic oil, *Zataria multiflora* Boiss., *Origanum vulgare*, *Cinnamomum zeylanicum*, carvacrol, and cinnamaldehyde [[43], [44], [45], [46]].

Liu from Yangzhou University, China, ranked third with 19 articles and 1,030 total citations. Much of his research focused on the properties and characteristics of antioxidant and antimicrobial packaging films based on chitosan, polysaccharides, and nanoparticles applied to various food products [[47], [48], [49]]. Liu with D. Yun et al. [50,51] has also extensively researched the utilization of natural pigments derived from food wastes as active components in active packaging.

### 3.6 Most cited article on “active food packaging”

Table 4 shows the 10 articles with the highest number of citations published between 2000 and 2021 related to active food packaging. The overall number of citations of the 10 articles ranged from 320 to 1,239, each published in seven different journals. The most cited article was written by Appendini et al. (1,239 total citations), entitled “Review of antimicrobial food packaging” published in 2002 in *Innovative Food Science and Emerging Technologies* with 59.00 average citations/year. The article was widely cited as it was a review and broadly discussed the types of antimicrobial polymers developed for food contact such as the addition of antimicrobial volatiles to sachets/pads, incorporation of antimicrobial compounds into polymers, through coating methods, and immobilization of antimicrobials in polymers through cross-linking. The article also examined the commercial application of antimicrobial packaging in food and effectiveness of antimicrobial compounds and the future outlook of antimicrobial packaging [52].

**Table 4:** The top-ten article cited in “Active Packaging for Food Packaging”.

SCR	Authors	Title	Year	Journal	Cited by	TC per year
1st	Appendini, P. et al.	Review of antimicrobial food packaging	2002	Innovative Food Science and Emerging Technologies	1,239	59.00
2nd	Jamshidian, Majid et al.	Poly-Lactic Acid: Production, applications, nanocomposites, and release studies	2010	Comprehensive Reviews in Food Science and Food Safety	951	73.15
3rd	Quintavalla, S. et al.	Antimicrobial food packaging in the meat industry	2002	Meat Science	689	32.81
4th	Ojagh, Seyed Mahdi et al.	Development and evaluation of a novel biodegradable film made from chitosan and cinnamon essential oil with a low affinity toward water	2010	Food Chemistry	569	43.77
5th	Rhim, Jong-whan et al.	Natural biopolymer-based nanocomposite films for packaging applications	2007	Critical Reviews in Food Science and Nutrition	558	34.88
6th	Cha, D.S. et al.	Biopolymer-based antimicrobial packaging: A review	2004	Critical Reviews in Food Science and Nutrition	522	27.47
7th	Seydim, A.C. et al.	Antimicrobial activity of whey protein-based edible films incorporated with oregano, rosemary, and garlic essential oils	2006	Food Research International	491	28.88
8th	Ozdemir, Murat et al.	Active food packaging technologies	2004	Critical Reviews in Food Science and Nutrition	373	19.63
9th	Azeredo, Henriette M.C. et al.	Nanocellulose in bio-based food packaging applications	2017	Industrial Crops and Products	329	54.83
10th	Mihindukulasuriya, S.D.F. et al.	Nanotechnology development in food packaging: A review	2014	Trends in Food Science and Technology	320	35.56

The second most cited article was written by Jamshidian et al. (951 total citations) titled “Poly-Lactic Acid: Production, Applications, Nanocomposites, and Release Studies” published in 2010 in *Comprehensive Reviews in Food Science and Food Safety* with 73.15 average citations/year. The article was a review that specifically discussed PLA derived from renewable and biodegradable sources. The article extensively reviewed the steps in the production process, general properties, applications, processing technologies, modifications and degradability, biodegradability, and recyclability of PLA polymers [53].

Quintavalla et al. [54] (689 total citations), Cha and Chinnan [55] (522 total citations), and Seydim and Sarikus [56] (491 total citations) discussed the characteristics of antimicrobial active packaging systems. Azeredo et al. [57] (329 total citations) and Mihindukulasuriya et al. [58] (320 total citations) specifically studied the development of nanotechnology, such as nanocellulose as a component material in the manufacture and application of food packaging.

### 3.7 Bibliographic coupling analysis

**Fig. 4:**
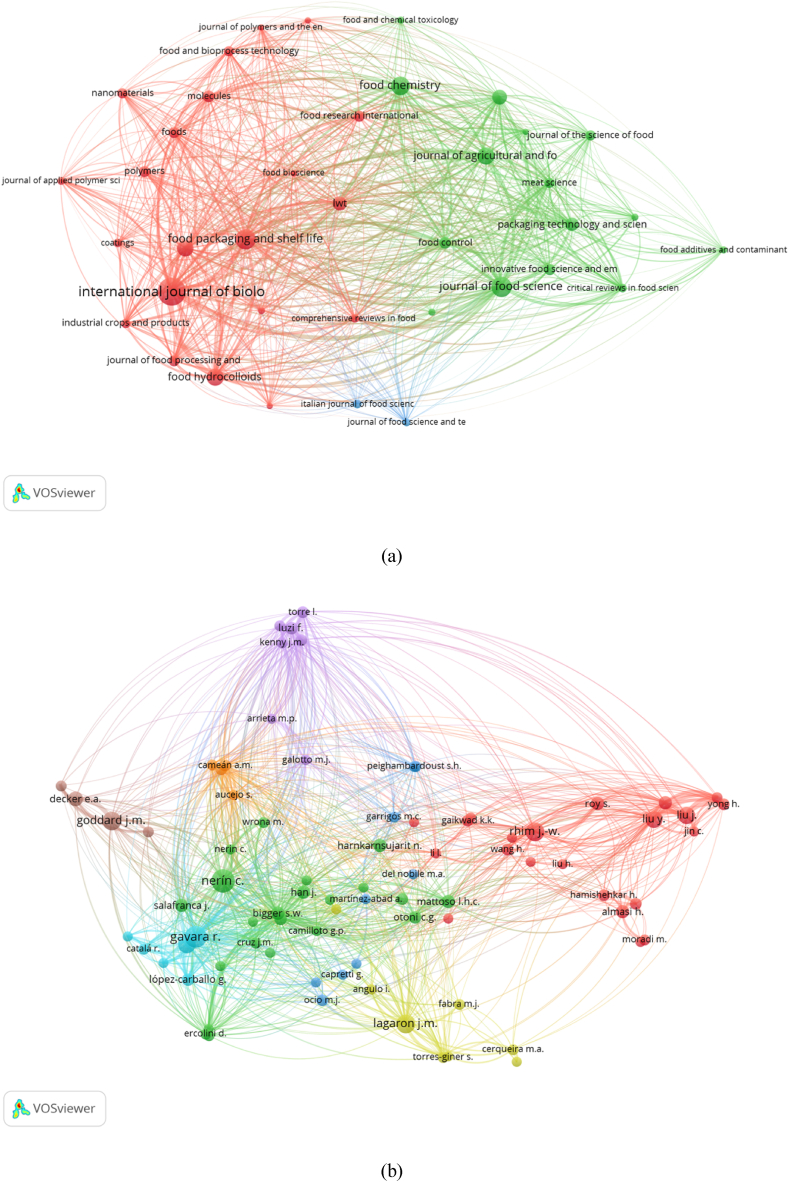

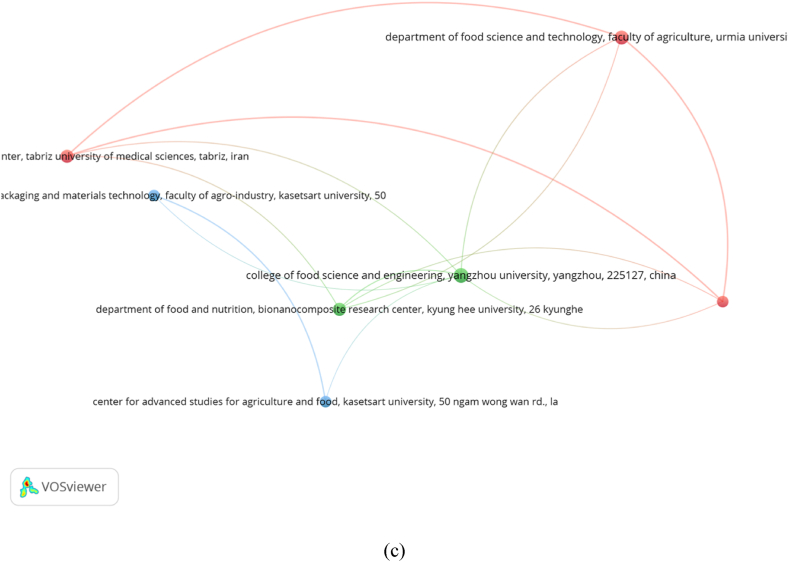
Bibliographic coupling analysis of (a) journals, (b) authors, and (c) institutions.

In this study, the full computation approach in VOSviewer was used to perform bibliographic coupling of journals, authors, and institutions. For the bibliographic coupling analysis, we selected a minimum of five articles and 38 of 189 journals that met the criteria. The strength of each journal's bibliographic coupling links to other sources is visualized in Fig. 4a. The 38 journals were divided into three clusters. The largest relationship of the first cluster (red) was with *the International Journal of Biological Macromolecules,* which had the highest total link strength of 9,525 with 84 articles and 3,584 total citations. Based on the total link strength, the *International Journal of Biological Macromolecules* showed the largest relationship with other journals, which was also in line with the largest total citations. The second cluster (green) was the *Journal of Food Science,* which had the highest total link strength of 6,000, 44 articles, and 1,243 citations. The third cluster (blue) belonged to the *Journal of Food Science and Technology* with the highest total link strength of 602, 7 articles, and 210 total citations.

Similarly, Fig. 4b shows the collaboration between the main authors of the published works. From the analysis results with the minimum number of authors set at 5, 82 of 2,940 total authors met the criteria. The 82 authors are classified into eight clusters marked with different colors. Gavara had the largest international collaboration network, with the highest total link strength of 5,696 with 24 articles, followed by Hernández-Muñoz with 4,566 total link strengths with 16 articles. The top two authors, although coming from different institutions, are still from the same country, Spain. The third author with a large collaboration network was Liu from Yangzhou University, China, with 4,463 total link strength and 14 articles.

Furthermore, for the analysis of bibliographic institutions, 7 of 1,880 institutions met the threshold (Fig. 4c). The seven institutions were classified into three clusters. Tabriz University of Medical Sciences had the highest total link strength of 610 with seven articles and was followed by Urmia University and Islamic Azad University with similar total link strength of 604 consisting of nine and five articles respectively.

### 3.8 Collaboration network analysis

**Fig. 5:**
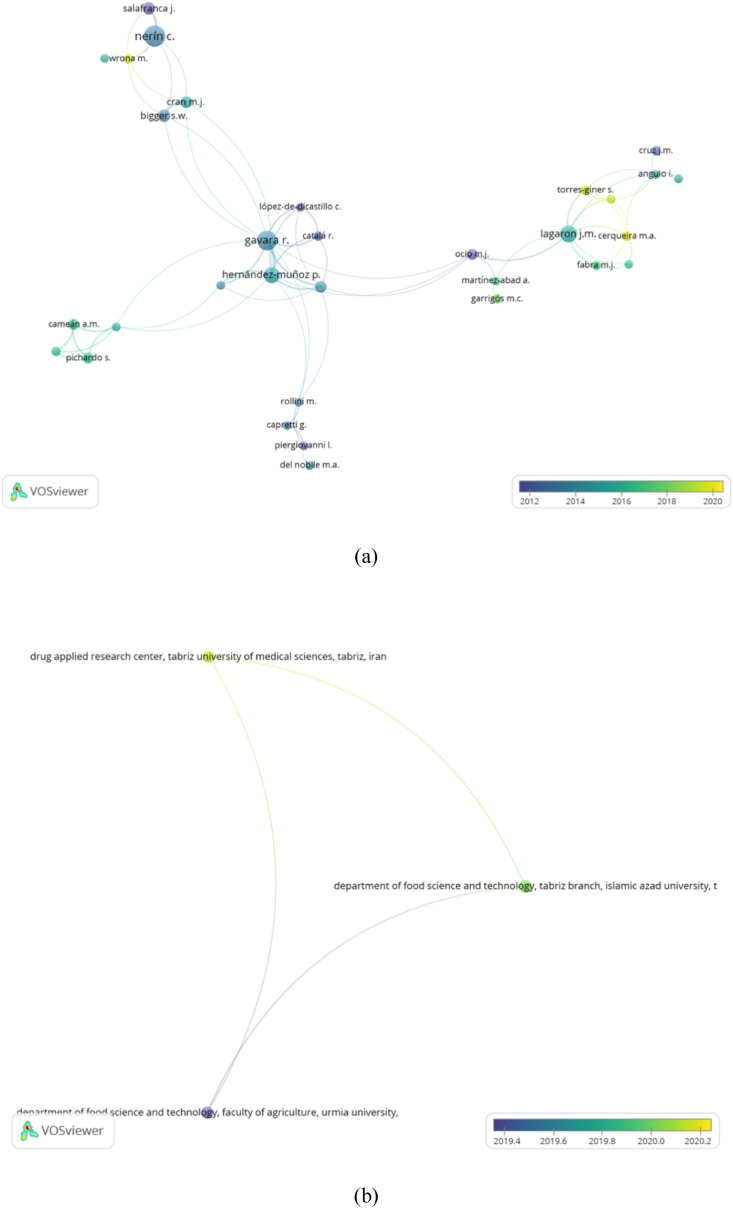

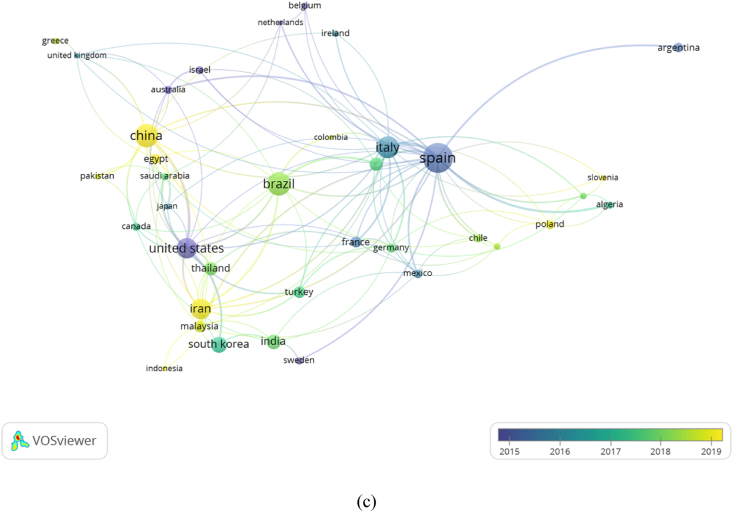
Collaborative network analysis of (a) authors, (b) institutions, and (c) countries.

In this study, a full-count approach in VOSviewer was used to analyze co-authors, institutions, and countries. The maximum number of authors per article was set at 20, and approximately 2,940 of them met the criteria. The minimum number of documents per author is set for 5 times out of 2,940 total authors, and only 82 authors met the analysis. Fig. 5a shows the relationship between authors.

Moreover, the maximum number of institutions was set at 25 per article. The minimum number of articles and citations obtained by 7 of 1,880 universities that met the criteria was set at 5 and 0, respectively. Furthermore, only 3 of 7 institutions were connected. They included Urmia University, Islamic Azad University, and Tabriz University of Medical Sciences. Fig. 5b shows the relationship between these institutions.

Furthermore, the maximum number of countries was set at 25 per article. The minimum number of articles and citations obtained by 37 of 69 states that met the criteria were set at 5 and 0, respectively. However, these 37 countries were grouped into 10 clusters based on the level of joint co-occurrence. States that have the same color (in one cluster) were interconnected. The size of a country's circle increases with the number of research articles produced and the scale of collaboration with the thickness of the connecting line (literature). This connecting line, called link strength, was used to measure the degree of cooperation between the two terms. Iran, Malaysia, and Indonesia tended to take part in joint publications. Based on Fig. 5c, the top three institutions with the highest total link strength are Spain, Italy, and USA having 81, 46, and 44 with 160, 88, and 77 articles, respectively. Furthermore, Australia, Israel, Netherlands, Belgium, Sweden, USA, Spain, France, and Italy published articles related to active food packaging that used to be very fast between 2015 and 2016. This topic started to gain massive discussion in 2021 in China, Iran, Egypt, Poland, and Slovenia.

### 3.9 Co-occurrence analysis

**Fig. 6:**
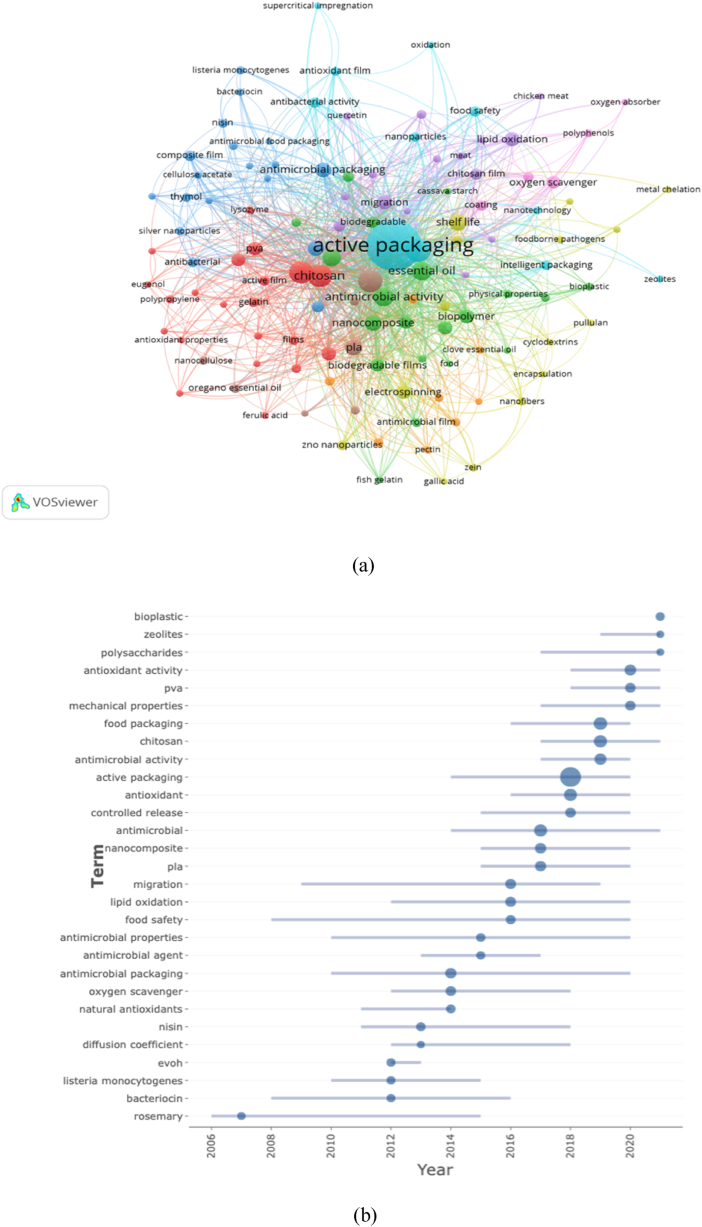
Cluster of keywords (a), development of keywords based on studies on active food packaging between 2000 and 2021 (b).

Co-occurrence analysis is used to pinpoint gaps and current trends to track developments in scientific research [[59], [60], [61]]. Fig. 6 shows the co-occurrence of author keywords checked using VOSviewer. The circle size indicated the publications that appear, whereas the distance provided an estimate of the relationship between frequent terminologies. This co-occurrence analysis used the full-count method. The minimum keyword occurrence is set for 5 times out of 1,775 total keywords, and only 120 keywords that met the analysis criteria were divided into nine clusters. Table 5 lists the top 15 keywords ranked by the total occurrences and link strength. Fig. 6a shows the grouping of terms frequently used by authors in works related to the bibliometric search for the past 21 years. Generally, the frequent keywords were active packaging, food packaging, antimicrobials, antioxidants, and chitosan having 445, 85, 73, 71, and 74 occurrences and 835, 199, 198, 181, and 167 total link strengths, respectively.

**Table 5:** The most used keywords (rank based on total link strength).

SCR	Keywords	Occurrences	Total link strength
1	Active packaging	445	835
2	Food packaging	85	199
3	Antimicrobial	73	198
4	Antioxidant	71	181
5	Chitosan	74	167
6	Antimicrobial activity	49	111
7	Essential oil	49	101
8	Antioxidant activity	42	93
9	Nanocomposite	35	92
10	PLA	33	76
11	Shelf life	36	75
12	Biopolymer	26	67
13	Edible file	21	65
14	migration	27	61
15	Active food packaging	29	58

Fig. 6b shows the development of keywords based on the studies of active food packaging between 2000 and 2021. From 2005 to 2015, bioactive compounds in rosemary were actively investigated. The study examined publications between 2008 and 2020 that focused on food safety and the development of packaging that has the main function as an antimicrobial for food safety between 2010 and 2020. Meanwhile, active packaging was hotly discussed in 2014. Bioplastics, zeolites, and polysaccharides are still frequently discussed presently. This is in line with the replacement of polymers made from petroleum with those that have a more positive environmental effect. Therefore, natural biopolymers are a choice for food packaging materials because of their biodegradable qualities that address current environmental challenges [[62], [63], [64]]. Polysaccharides, which were derived from abundant renewable resources, such as starch, chitosan, alginate, and/or cellulose, have been studied extensively [[65], [66], [67], [68]]. This is in line with the results of Marzano-Barreda et al. [69] who said that food packaging made from cassava starch and biodegradable containing synthetic zeolite can increase the shelf life of fresh broccoli.

### 3.10 Application of active food packaging

**Fig. 7:**
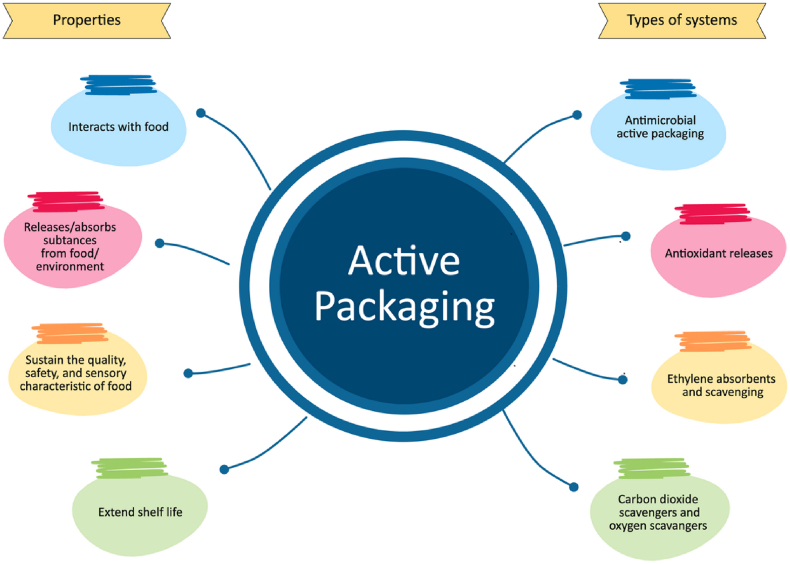
Active packaging classification and their main properties.

Consumer preference for fresh and safe food products has led to the exploration of advanced techniques to maintain product quality and shelf life [70,71]. However, the highly perishable properties of the product due to microbial damage and high moisture content often limit its storage potential. To mitigate these issues, effective packaging approaches such as the utilization of active packaging have been developed to create barriers against gases, moisture, and microbes, increasing their shelf life [[72], [73], [74]]. Classifications of active packaging that can be applied to food products are illustrated in Fig. 7. The utilization of antioxidants and antimicrobials has emerged as a promising strategy in the food industry, commonly employed as active ingredients for the production of coatings and films [[75], [76], [77]].

Numerous studies have indicated the potential of incorporating antioxidant and antimicrobial agents into food packaging to enhance the quality and safety of food products such as fruits, vegetables, meat, and other consumables. In this context, Table 6 summarizes the applications of active packaging in the form of films/coatings on various food types. For example, PVA-based films combined with grape seed flour as active substances were evaluated for raisin storage [78]. Another example is the application of alginate-based active packaging with the addition of aloe vera/garlic oil on fresh tomatoes [79]. Additionally, the effect of combining a chitosan/pectin/gelatin composite with metal nanoparticles/lemongrass essential oil as an active component on the quality of raspberries was also examined [80].

**Table 6:** Application and function of bioactive components in active packaging and their effect on food products.

Biopolymers	Active substances	Plasticizer/additives	Film/coating	Application	Effects	Ref
Chitosan	White turmeric	1% glycerol	Film	NA	White turmeric incorporated chitosan exhibited extensive antimicrobial and antioxidant properties; and excellent mechanical properties	[76]
Gum arabic/cellulose nanocrystals	Pomegranate peel extract/grape skin extract/red pitaya peel extract	3% (w/v) glycerol	Film	Raspberry	Incorporation pomegranate peel extract improved the flexibility and light resistance of films and effectively enhanced the antioxidant activities	[81]
Carrageenan/agar	Tea tree oil/zinc sulfide nanoparticles	1.2 g glycerol	Film	NA	The mechanical, thermal, and water vapor barrier properties of the binary composite films exhibited minimal changes and also showed moderate antimicrobial and antioxidant activity	[82]
Nanochitosan	*Cuminum cyminum* essential oil	Glycerol	Coating	Sardine fillet fish	Coatings reduced lipid oxidation, microbial growth, and improved sensory attributes and had better effects on quality attributes of Sardine fillet fish.	[77]
Polyvinyl alcohol (PVA)	Grape seed flour	2% (w/v) citric acid	Film	Raisin	film contain higher levels of phenolic content and antioxidant activity values and film can act in the preservation of raisin	[78]
gelatin/carrageenan	Enoki mushroom-derived carbon dots	NA	Film	NA	Improved physical and functional properties, and exhibited strong antioxidant activity of the film	[83]
poly (butylene succinate) (PBS)	Curcumin/carvacrol	NA	Film	NA	Carvacrol showed higher influence on antimicrobial activity (*E. coli*.), whereas curcumin showed higher influence on the antioxidant properties of the films.	[84]
Agarose/agaropectin	Bacteriocin (cell-free extract)	glycerol	Film	Curd Cheese	Incorporation of bacteriocin in film improved food barrier characteristics and contributed to the reduction of contamination of a food	[85]
Polypropylene	Layered double yydroxides (an-timicrobial sorbate anion)	NA	Coating	Bread	Prolonged shelf life of bread with higher sensorial attributes up to 12 days of storage at ambient temperature.	[86]
Chitosan	Pomegranate peel extract	30% w/w glycerol	Film	NA	Improved the antioxidant and antimicrobial properties of the chitosan film Film showed excellent ultraviolet–visible light barrier capacity	[87]
Alginate	Aloe vera/garlic oil	40% wt glycerol	Coating	Tomatoes	Improved barrier, UV-shielding, and antimicrobial properties of films Extend shelf life of tomatoes	[79]
poly (lactic acid) (PLA)/poly (butylene adipate terephthalate) (PBAT)	Carvacrol essential oil	0%,2% and 5% carvacrol	Film	Bread/butter cake	Films with incorporation carvacrol showed delayed growth and sporulation of *Penicillium* sp. and *Rhizopus* sp Carvacrol plasticized reducing relaxation temperature	[88]
Carboxymethyl cellulose	*Satureja hortensis* essential oil	50% v/w glycerol	Coating	NA	Incorporation essential oil of films displayed better water vapor barrier and lower solubility film presented good inhibition against the tested bacteria	[89]
Chitosan/pectin/gelatin	Metal nanoparticles/Lemongrass essential oil	Glycerol	Coating	Raspberries	Prolonged the shelf life of raspberries from 4 to 8 days The combination and synergistic effect of metal nanoparticles and lemongrass essential oil led to the best antimicrobial properties	[80]
PBAT/PLA	Carvacrol, citral and α-terpineol essential oils	carvacrol, citral and α-terpineol essential oils	Film	Shrimp	Films containing EOs showed delayed microbial growth, reduced lipid oxidation and drip loss	[75]
Starch	Sunflower oils	NA	Coating	NA	The thermal release of oil shows strong sensitivity of the release profiles towards the composition of encapsulated oil	[90]

Edible films and coatings may improve the quality and extend the shelf life of various food products. This is due to their ability to inhibit lipid oxidation, prevent moisture loss, prevent discoloration, and inhibit contamination caused by bacteria, molds, and yeasts [71,91]. In addition, these coatings can trap volatile flavor compounds, thereby maintaining the sensory properties of the ingredients. Kang et al. [81] successfully maintained the quality and shelf life of raspberries by incorporating pomegranate peel extract/grape skin extract/red pitaya peel extract into gum Arabic-cellulose nanocrystal film-forming matrix, and the film with pomegranate peel extract showed the best effect on the preservation of raspberries. Sardine fillets coated with *Cumino cyminum* L. essential oil with nano chitosan base enabled the control of microbial agents on the surface of the samples and showed a significant reduction in the number of microbes compared with the uncoated ones, thus extending the shelf life by 16 days at 4 °C [77]. The incorporation of carvacrol essential oils into PLA films showed a delay in mold growth and sporulation of *Penicillium* sp. and *Rhizopus* sp. [88].

### 3.11 Challenges, future perspective, and limitation

Accumulating scientific evidence shows that active packaging is a very promising research topic that must be further explored. For several reasons, further research on active food packaging can contribute to the development of the food industry because active packaging can protect the product and maintain its freshness, providing a relatively long shelf life. Active packaging can be a trend for food science and technology, with its diverse applications in the future. To improve and develop this research trend, knowledge about the characteristics, properties, toxicity, and ability of active packaging is important to protect the packaged material that is more productive and efficient for future applications.

Given that food products are mostly perishable, packaging parameters are highly dependent on the specific product. Therefore, to achieve the optimum performance or capacity of the desired active packaging system, a product-tailored strategy should be applied. All contributing factors such as the physicochemical and physiological properties of the food, packaging size, storage conditions, and effect on the environment must be considered. Recent research trends, as indicated by keyword analysis, demonstrate a growing interest in investigating the application of natural polymers like polysaccharides and their derivatives in active packaging because they are easily degraded compared to conventional plastics, which has a positive impact on the environment. Thus, environmentally friendly active packaging (bioplastics) can contribute to 17 sustainable development goals for 2030. The implementation of active packaging systems on an industrial scale must also consider the cost of implementing the technology in line with the benefits derived from the product, and regulatory and legislative issues must be addressed while ensuring widespread consumer acceptance. Successful collaboration between research institutions and industry is required to overcome these challenges. Nonetheless, recent trends over the past 21 years as discussed in this review can provide scientists, the public, and packaging industry institutions with a better understanding of the potential and benefits of active packaging technologies, facilitating their large-scale commercialization.

Notwithstanding the contribution of this study, it has some limitations. When interpreting the results of the bibliometric analyses, the methodological limitations must be acknowledged. These limitations are inherent and cannot be completely avoided because of the absence of a perfect and all-encompassing search strategy. In addition, the search methodology employed is also subject to limitations. Specifically, only literature indexed in the Scopus database was used, which means that articles published in non-Scopus-indexed journals were not evaluated. Although Scopus is a widely trusted and comprehensive database for bibliometric analyses [24,25], these limitations should be taken into consideration. Potential future investigations could incorporate additional databases to verify the generalizability of our findings. Nonetheless, the majority of the records cataloged in prominent bibliographic databases such as Scopus and WoS exhibit dual-indexing within both databases [92]. Despite these limitations, the findings of this study can contribute to a valid discussion of the scientific literature on the topic of “application of active food packaging.”

## 4 Conclusion

Throughout the bibliometric study, the number of research articles on active food packaging has increased since 2000, which makes it a research area with outstanding scientific production. Based on the scientific mapping of global research on active packaging from 2000 to 2021, a total of 857 articles were found in the Scopus database, written by 2,941 authors from 58 countries. In terms of publication distribution by country, Spain had the highest number of published articles, with a large network of collaborations. In addition, the *International Journal of Biological Macromolecules* was the most relevant journal related to the subject with a total of 84 articles. A high collaboration network was also found between authors, with the strongest collaboration network between Spain, Italy, and the USA.

From a systematic analysis of research published over the past 21 years, several insights and future study directions on the application of active packaging in food packaging were identified. Based on recent term (keyword) trends in time, the concepts of antimicrobial packaging and biodegradability are currently frequently reviewed and discussed among researchers, for example, the use of environmentally friendly packaging (bioplastic) based on polysaccharides and nanoparticles (zeolite). These new terms may be developed or collaborated with other keywords to create breakthroughs in the future. However, further studies related to the manufacturing process and toxicity studies are still needed to ensure that the resulting packaging is considered safe for packaged food products. Based on this, the application of active food packaging shows a promising trend for researchers and the packaging industry in the future.

## Author contribution statement

All authors listed have significantly contributed to the development and the writing of this article.

## Data availability statement

Data will be made available on request.

## Declaration of competing interest

The authors declare that they have no known competing financial interests or personal relationships that could have appeared to influence the work reported in this paper.
